# Supplementary material for: Molecular Cloning of the B4GALNT2 Gene and Its Single Nucleotide Polymorphisms Association with Litter Size in Small Tail Han Sheep
Source: Animals (Basel). 2018 Sep 20;8(10):160. doi: 10.3390/ani8100160 (PMC6210199; doi:10.3390/ani8100160)
Supplement: Supplementary file 1 [file animals-08-00160-s001.zip › Supplementary file/Supplementary file 1.pdf]

Supplementary file 1 Sequences of the primers used in the amplifications

Primers	Primer sequence (5'-3')	Usage
P1	F: CCTCAACAAAGGCTGGTTCA<br>R: GCATGGCATGTGATAGGGAC	g.36938224T>A detection
P2	F: TGCTCCGGGGTGTTTTCC<br>R: CTCTGCAGTGTTGGTGTGTC	g.37034573A>G detection
P3	F: CCGACCACCCATTTCTTCT<br>R: GGTTTGCTTTCCTGGTGTGT	DLX3:c.*803A>G detection
P4	F: TCAGCATCTACAAAGCACCACTC<br>R: TCACATTCACAATCAGCACAGGT	g.36971115C>T detection
P5	F: TTTAGCACCACCAGAGTTTGAGG<br>R: ACCCAAACAGAGGCAACACAG	g.36946470 C>T,<br>g.36946465 G>A detection
P6	F: GCATTTAGTCTTGGCGTCTCG<br>R: GCTGGTGGTGTTTTTCTGGGT	g.36942215T>C detection
P7	F: AAACCCTCAGAATGACGCAAG<br>R: TCAGAGGAGAATCCCACACCC	g.36933082C>T,<br>g.36933070G>A detection
P8	F: TGGCAGTGTGCTTGGAAATGT<br>R: AAGTAGGAACAGCCTGACGGA	g.36930089T>G detection
P9	F: ACCACTCCTTCAGCCTAGTG<br>R: TCATCGTCCACCCAGAGAAC	*B4GALNT2*-conservative sequence
P10	F: CAGACTGAACTCCCTGCGGTGA<br>R: CACATCCAGTTCGGTCTTCTC	*B4GALNT2*-conservative sequence
P11	F: AAGATTGAGGTGCTGGTGGA<br>R: TGCCCTGATCAGTGATTGGT	*B4GALNT2*-conservative sequence
P12	F: CAGCCCTGGAGAAGACCTAC<br>R: CCCACGCTCTGATCCTCTAA	*B4GALNT2*-conservative sequence
P13	F: CTTGAGGAAGTGGCAGTCTG<br>R: GCCACTGCGCTTACAAAGTA	*B4GALNT2*-conservative sequence
P14	F: ATGGGATGCAAGAAGGTGGA<br>R: TTTTGACCTACCGTGGCTGT	*B4GALNT2*-conservative sequence
5'GP1	AGAGGCTCTTGATGTGTTTCTCAGGGAG	*B4GALNT2* - 5'RACE
5'GP2	CCCAGGGCCAAGAATAAGACGGACATC	*B4GALNT2* - 5'RACE
3'GIP	TGGGATGCAAGAAGGTGGAGGGCAGAG	*B4GALNT2* - 3'RACE
3'GOP	GATTCTCCCACTGCTGCGCTGCCTTTT	*B4GALNT2* - 3'RACE
P15	F: CCTGAAAGCTTCTCTGGGGA<br>R: CCGGCTACTGGTCAAAATGG	*B4GALNT2* - qRT
P16	F:CCAACCGTGAGAAGATGACC<br>R:CCCGAGGCGTACAGGGACAG	*β-actin* - qRT
