# Supplementary material for: Molecular Cloning of the B4GALNT2 Gene and Its Single Nucleotide Polymorphisms Association with Litter Size in Small Tail Han Sheep
Source: Animals (Basel). 2018 Sep 20;8(10):160. doi: 10.3390/ani8100160 (PMC6210199; doi:10.3390/ani8100160)
Supplement: Supplementary file 1 [file animals-08-00160-s001.zip › Supplementary file/Supplementary file 4.pdf]

Supplementary file 4: Six high similarity protein sequences of ovine B4GALNT2 for motifs detection

> B4GALNT2_Ovis aries, NP_001305005.1
MTSFGSRYLWILKMSVLFLALGIVLFMFRSVSLYSEFSIYKAPLLQPSVAAGMLKLLPEKHI
KSLFTHDGIWLFPKNQCKCEAWKLQQSYYFENAYGQTELPAVKMRRQAEFEHFQRREGL
PRPPPLLAQANLPFGYPIHGVEVMPLHTIPIPGLQFEGPDAPIYKVTLKASLGTLNTLADTP
DTVVQGRGQKELTILTSSRTVLNFILQHVTYTSTEYQHPRVDVVSLESNSSVAKFPVTIRFP
VMPKLYDPGPERKLRNLVSIATKTFLRPHKLKTMLQSIRVYYPDLTVIVADDSKEPLEINDS
YVEYYTMPYGKGWFAGRNLAISQVTTKYVLWVDDDFLFNDKTKIEVLVDVLEKTELDV
VGGSVLGNVFQFKLFLEHSKNGDCLHRRTGSFGPLDGFPNCVVTSGVVNFFLAHTERLQR
VGFDPRLHRVAHSEFFIDGLGSLLVGSCSDVIIGHQPHSSVADPELAALEKTYRRYRANTN
DKVQFKLALHYFKNHLQCTT
>B4GALNT1_Rattus norvegicus, AAH81799.1
MRLDRRALYALVLLLACASLGLLYASTRDAPGLPNPLALWSPPQGPPRLDLLDLATEPRYA
HIPVRIKEQVVGLLAQNNCSCESSGGRFALPFLRQVRAIDFTKAFDAEELRAVSISREQEYQ
AFLARSRSLADQLLIAPANSPLQYPLQGVEVQPLRSILVPGLSLQEASVQEIYQVNLIASLG
TWDVAGEVTGVTLTGEGQSDLTLASPILDKLNRQLQLVTYSSRSYQANTADTVRFSTKGH
EVAFTILIRHPPNPRLYPPSSLPQGAQYNISALVTVATKTFLRYDRLRALIASIRRFYPTVTIVI
ADDSDKPERISDPHVEHYFMPFGKGWFAGRNLAVSQVTTKYVLWVDDDFVFTARTRLEK
LVDVLERTPLDLVGGAVREISGYATTYRQLLSVEPGAPGFGNCLRQKQGFHHELAGFPNC
VVTDGVVNFFLARTDKVRQVGFDPRLNRVAHLEFFLDGLGSLRVGSCSDVVVDHASKVK
LPWTSKDPGAELYARYRYPGSLDQSQVAKHRLLFFKHRLQCMTAE
>B4GALNT1_Mus musculus, AAH57199.1
MRLDRRALYALVLLLACASLGLLYSSTRNAPSLPNPLALWSPPQGPPRLDLLDLAPEPRYA
HIPVRIKEQVVGLLAQNNCSCESKGGSLPLPFLRQVRAVDLTKAFDAEELRAVSVAREQEY
QAFLARSRSLADQLLIAPANSPLQYPLQGVEVQPLRSILVPGLSLQEASVQEIYQVNLSASL
GTWDVAGEVTGVTLTGEGQPDLTLASPVLDKLNRQLQLVTYSSRSYQANTADTVRFSTK
GHEVAFTILVRHPPNPRLYPPSSLPQGAEYNISALVTIATKTFLRYDRLRTLIASIRRFYPTVTI
VIADDSDKPERISDPHVEHYFMPFGKGWFAGRNLAVSQVTTKYVLWVDDDFVFTARTRL
EKLVDVLEKTPLDLVGGAVREISGYATTYRQLLSVEPGAPGLGNCFRQKQGFHHELVGFPS
CVVTDGVVNFFLARTDKVRQVGFDPRLNRVAHLEFFLDGLGFLRVGSCSDVVVDHASKV
KLPWTAKDPGAETYARYRYPGSLDQSQVAKHRLLFFKHRLQCMTAE
>B4GALNT2_Homo sapiens, NP_703147.2
MGSAGFSVGKFHVEVASRGRECVSGTPECGNRLGSAGFGALCLELRGADPAWGPFAAHG
RSRRQGSRFLWLLKILVIILVLGIVGFMFGSMFLQAVFSSPKPELPSPAPGVQKLKLLPEERL
RNLFSYDGIWLFPKNQCKCEANKEQGGYNFQDAYGQSDLPAVKARRQAEFEHFQRREGL
PRPLPLLVQPNLPFGYPVHGVEVMPLHTVPIPGLQFEGPDAPVYEVTLTASLGTLNTLADV
PDSVVQGRGQKQLIISTSDRKLLKFILQHVTYTSTGYQHQKVDIVSLESRSSVAKFPVTIRH
PVIPKLYDPGPERKLRNLVTIATKTFLRPHKLMIMLRSIREYYPDLTVIVADDSQKPLEIKDN
HVEYYTMPFGKGWFAGRNLAISQVTTKYVLWVDDDFLFNEETKIEVLVDVLEKTELDVV
GGSVLGNVFQFKLLLEQSENGACLHKRMGFFQPLDGFPSCVVTSGVVNFFLAHTERLQRV
GFDPRLQRVAHSEFFIDGLGTLLVGSCPEVIIGHQSRSPVVDSELAALEKTYNTYRSNTLTR
VQFKLALHYFKNHLQCAA
>B4GALNT2_Mus musculus, NP_032107.1

MTSSVSFASFRFPWLLKTFVLMVGLATVAFMVRKVSLTTDFSTFKPKFPEPARVDPVLKLL
PEEHLRKLFTYSDIWLFPKNQCDCNSGKLRMKYKFQDAYNQKDLPAVNARRQAEFEHFQ
RREGLPRPPPLLAPPNLPFGYPVHGVEVMPLHTILIPGLQYEGPDAPVYEVILKASLGTLNT
LADVPDDEVQGRGQRQLTISTRHRKVLNFILQHVTYTSTEYYLHKVDTVSMEYESSVAKF
PVTIKQQTVPKLYDPGPERKIRNLVTIATKTFLRPHKLKILLQSIRKYYPDITVIVADDSKEPL
EINDDYVEYYTMPFGKGWFAGRNLAISQVTTKYVLWVDDDFLFSDKTKIEVLVDVLEKT
ELDVVGGSVQGNTYQFRLLYEQTKNGSCLHQRWGSFQALDGFPGCTLTSGVVNFFLAHT
EQLRRVGFDPILQRVAHGEFFIDGLGRLLVGSCPGVIINHQVRTPPKDPKLAALEKTYDKY
RANTNSVIQFKVALQYFKNHLYCST
>B4GALNT1_Homo sapiens, NP_001469.1
MWLGRRALCALVLLLACASLGLLYASTRDAPGLRLPLAPWAPPQSPRRPELPDLAPEPRYA
HIPVRIKEQVVGLLAWNNCSCESSGGGLPLPFQKQVRAIDLTKAFDPAELRAASATREQEF
QAFLSRSQSPADQLLIAPANSPLQYPLQGVEVQPLRSILVPGLSLQAASGQEVYQVNLTASL
GTWDVAGEVTGVTLTGEGQADLTLVSPGLDQLNRQLQLVTYSSRSYQTNTADTVRFSTEG
HEAAFTIRIRHPPNPRLYPPGSLPQGAQYNISALVTIATKTFLRYDRLRALITSIRRFYPTVTV
VIADDSDKPERVSGPYVEHYLMPFGKGWFAGRNLAVSQVTTKYVLWVDDDFVFTARTRL
ERLVDVLERTPLDLVGGAVREISGFATTYRQLLSVEPGAPGLGNCLRQRRGFHHELVGFPG
CVVTDGVVNFFLARTDKVREVGFDPRLSRVAHLEFFLDGLGSLRVGSCSDVVVDHASKLK
LPWTSRDAGAETYARYRYPGSLDESQMAKHRLLFFKHRLQCMTSQ
