# Supplementary material for: Molecular Cloning of the B4GALNT2 Gene and Its Single Nucleotide Polymorphisms Association with Litter Size in Small Tail Han Sheep
Source: Animals (Basel). 2018 Sep 20;8(10):160. doi: 10.3390/ani8100160 (PMC6210199; doi:10.3390/ani8100160)
Supplement: Supplementary file 1 [file animals-08-00160-s001.zip › Supplementary file/Supplementary file 5.pdf]

Supplementary file 5: Homologous mRNA sequences of ovine B4GALNT2 for phylogenetic tree construct

>B4GALNT2_Ovis aries, NM_001318076.1
GGCTGGACAATCGGCTGGGAGGCTGCCCGGCTCCACCAGCCCAGGGCGTCCGAGGCG
GCGGGATTCGTGATGACTTCGTTCGGCTCTAGATATCTGTGGATCCTCAAGATGTCCGT
CTTATTCTTGGCCCTGGGCATTGTATTATTTATGTTCAGAAGTGTGTCCCTGTATTCAGAA
TTCAGCATCTACAAAGCACCACTCCTTCAGCCTAGTGTGGCTGCCGGAATGCTGAAGC
TTCTCCCTGAGAAACACATCAAGAGCCTCTTTACCCACGATGGAATCTGGCTCTTCCCG
AAAAACCAGTGCAAATGTGAAGCCTGGAAACTTCAGCAAAGCTATTACTTTGAAAATG
CTTATGGCCAGACTGAACTCCCTGCGGTGAAAATGAGGAGACAAGCTGAATTTGAACA
CTTTCAGAGAAGAGAAGGGCTGCCTCGCCCACCGCCCCTGCTGGCTCAGGCCAACCT
CCCCTTTGGGTACCCTATCCACGGGGTGGAAGTGATGCCCCTGCACACAATTCCCATCC
CAGGTCTCCAGTTTGAAGGACCAGACGCTCCCATCTATAAGGTCACCCTGAAAGCTTC
TCTGGGGACACTGAACACCCTCGCTGACACCCCAGACACTGTGGTGCAGGGCAGAGG
CCAGAAGGAGCTGACCATTTTGACCAGTAGCCGGACGGTTTTGAATTTCATCCTCCAG
CACGTGACATACACCAGCACAGAGTACCAGCACCCCAGGGTGGATGTGGTGAGTCTG
GAGTCCAACTCCTCAGTGGCCAAGTTTCCAGTGACCATCCGCTTTCCTGTTATGCCTAA
GTTATATGACCCTGGACCAGAGAGGAAGCTCAGAAACCTGGTGTCCATTGCCACCAAA
ACTTTCCTCCGTCCCCACAAGCTCAAGACCATGCTCCAGAGCATTCGTGTGTATTACCC
AGACTTGACCGTGATCGTCGCTGATGACAGCAAGGAGCCCCTGGAAATTAATGACAGC
TACGTGGAGTATTACACCATGCCCTACGGCAAGGGTTGGTTTGCTGGTAGGAACCTGG
CCATATCTCAGGTTACCACCAAATACGTTCTCTGGGTGGACGATGACTTTCTCTTCAATG
ACAAGACCAAGATTGAGGTGCTGGTGGATGTCCTAGAGAAGACCGAACTGGATGTGG
TAGGTGGCAGTGTGCTTGGAAATGTGTTCCAGTTTAAGCTGTTCCTGGAGCACAGCAA
GAATGGGGACTGTCTCCATCGGCGGACAGGATCTTTCGGGCCCCTGGACGGCTTCCCC
AACTGCGTGGTGACCAGCGGCGTCGTTAACTTCTTCCTGGCCCACACAGAGCGGCTCC
AAAGAGTTGGCTTTGACCCCCGCCTGCATCGAGTGGCTCACTCAGAGTTCTTTATTGAT
GGGCTCGGGAGTCTGCTCGTGGGGTCCTGCTCAGATGTGATTATAGGTCACCAGCCCC
ATTCTTCAGTGGCGGACCCAGAGCTGGCAGCCCTGGAGAAGACCTACCGCAGATACCG
GGCCAACACCAATGATAAGGTCCAGTTCAAGCTAGCTCTCCACTACTTCAAGAACCAT
CTCCAATGTACCACATAAAGATGCTTGGACTTTGGCAGAGTGCTAGGTCTGGCTCAGTT
ACTGTAGGTCATTAGAAATGGACCAATCACTGATCAGGGCAATGGCGATTGTATTAATA
ATAATTATTGTTTGGTGTCTTACCATTTGGGAATCACTGTACACACCTGATATGAAGAGC
TGACCCATTAGAAAAGACCCTAACGCTGGGAAAGATGGAAGGCAGGAGGAGAAGGG
GACGACAGAGGATGAGATGGTTAGATGGCATCACCGACTCGATGGACATGAGTTTGGG
TAAACTCCGGGAGTTTGCTGCGGTTCATGGGGTCACAAAGAGCTGGACACGACAGAG
TGACTGAACTGTACAAGTGAACTGTACATATAATGGTGGTGGTGGTGGTTTAGGCAATA
AGTCGTGTCCAACTTTCGTCTCACATCATCTCATGCAAAAGAGCACATGATGGACTGGT
ATCCTTGATCAAAAGGATCAAAAGGATCGGTATCCTCTGGGTCCTTGAGAGTTATTTGG
TGGGTGTTACATCACAGATCATCTCTAGCATCAACT

>B4GALNT2_Capra hircus, NM_001314262.1
ATGACTTCGTTCGGCTCTAGATATCTGTGGATCCTCAAGATGTCCGTCTTATTCTTGGCC
CTGGGCATTGTATTATTTATGTTCAGAAGTGTGTCCCTGTATTCAGAATTCAGCATCTAC
AAAGCACCACTCCTTCAGCCTAGTGTGGCTGCCGGAATGCTGAAGCTTCTCCCTGAGA

AACACATCAAGAGCCTCTTTACCCACGATGGAATCTGGCTCTTCCCGAAAAACCAGTG
CAAATGTGAAGCCTGGAAACTTCAGCAAAGCTACTACTTTGAAAATGCTTATGGCCAA
ACTGAACTCCCTGCGGTGAAAATGAGGAGACAAGCTGAATTTGAACACTTTCAGAGA
AGAGAAGGGCTGCCTCGCCCACCGCCCCTGCTGGCTCAGGCCAACCTCCCCTTTGGGT
ACCCCATCCACGGGGTGGAAGTGATGCCCCTGAACACAATTCCCATCCCAGGTGTCCA
GTTTGAAGGACCAGACGCTCCCATCTATAAGGTCACCCTGAAAGCTTCTCTGGGGACA
CTGAACACCCTCGCTGACACCCCAGACACTGTGGTGCAGGGCAGAGGCCAGAAGGAG
CTGACCATTTTGACCAGTAGCCGGACGGTTTTGAATTTCATCCTCCAGCACGTGTCATA
CACCAGCACAGAGTACCAGCACCCCAGGGTGGATGTGGTGAGTCTGGAGTCCAACTC
CTCAGTGGCCAAGTTTCCAGTGACCATCCGCTTTCCTGTTATGCCTAAGTTATATGACCC
TGGACCAGAGAGGAAGCTCAGAAACCTGGTGTCCATTGCCACCAAAACTTTCCTCCGT
CCCCACAAGCTCAAGACCATGCTCCGGAGCATTCGTGAGTATTACCCAGACTTGACTG
TGATCGTGGCTGATGACAGCAAGGAGCCCCTGGAAATTAATGACAGCCATGTGGAGTA
TTACACCATGCCCTACGGCAAGGGTTGGTTTGCCGGTAGGAACCTGGCCATATCTCAGG
TCACCACCAAATACGTTCTCTGGGTGGACGATGACTTTCTCTTCAATGAGAAGACCAA
GATTGAGGTGCTGGTGGATGTCCTAGAGAAGACCGAACTGGATGTGGTAGGTGGCAGT
GTGCTTGGAAATACGTTCCAGTTTAAGCTGTTCCTGGAGCACACTAAGAATGGGGACT
GTCTCCATCGGCGGACAGGATCTTTCGGGCCCCTGGATGGCTTCCCCAACTGCGTGGT
GACCAGCGGTGTCGTTAACTTCTTCCTGGCCCACACAGAGCGGCTCCAAAGAGTTGGC
TTTGACCCCCGCCTGCAGCGAGTGGCTCACTCAGAGTTCTTTATTGATGGGCTCGGGA
GTCTGCCCGTGGGGTCCTGCTCAGATGTGATTATAGGTCACCAGCCCCATTCTTCAGTG
GCGGACCCAGAGCTGGCAGCCCTGGAGAAGACCTACGGCAGATACCGGGCCAACACC
AATGATAAGGTCCAGTTCAAGCTAGCTCTCCACTACTTCAAGAACCATCTCCAATGTAC
CACATAA
>B4GALNT2_Bos taurus, XM_010816190.2
CTACCCCGGGTTGGTGCCTAGGGCTGGACAATCGGCTGGGAGGCTGCCGGGCTCCACC
AGCCCAGGGCGTCCGAGGCGGCGGGATTCATGATGACTTCGTTCGGCTCTAGATATCTG
TGGATCCTCAAGATGTCCGTCTTATTCTTGGCCCTGGGCATTGTGTTATTTATGTTCAGA
AGTGTGTACCTGTATTCAGAATTCAGCAGCTACAAAGCACCACTCCTTCAGCCTAGTCC
GGCTGCCGGAATGTTGAAGCTTCTCCCTGAGGAACATATCAAGACCCTCTTTACCCAC
GATGGAATCTGGCTCTTCCCGAAAAACCAGTGCAAATGTGAAGCCTGGATACATCGGC
AAAGATATAACTTTGAAAATGCCTATGACCAGACTGACCTCCCTGCGGTGAAAATGAG
GAGACAAGCTGAATTTGAACACTTTCAGAGGAGAGAAGGGCTGCCTCGCCCACCACC
CCTGCTGGCTCAGGCCAACCTCCCCTTTGGGTACCCGGTCCACGGAGTGGAGGTGATG
CCCCTGCACACAATTCCCATCCCGGGTCTCCAGTTTGAAGGACCAGACGCTCCCATCTA
TAAGGTCACCCTGAAAGCTTCTCTGGGGACACTGAACACCCTCGCTGACACCCCAGAC
AGTGTGGTGCAGGGCCGAGGCCAGAAGCAGCTGACCATTTTGACCAGTAGCCGGAAG
GTTTTGAATTTCATCCTCCAGCACGTGACATACACCAGCACAGAGTACCAGCACCACA
GGGTGGATGTGGTGAGTCTGGAGTCCAACTCCTCAGTGGCCAAGTTTCCAGTGACCAT
CCGCTTTCCTGTTATGCCTAAGTTATACGACCCTGGACCAGAGAGGAAGCTCAGAAAC
CTGGTGTCCATTGCCACCAAAACTTTCCTCCGTCCCCACAAGCTCATGACCATGCTCCA
GAGCATTCGTGAGTATTACCCAGACTTGACCGTGATCGTGGCTGATGACAGCAAGGAG
CCCCTGAAAATTAATGACAGCCATGTGGAGTATTACACCATGCCCTACGGCAAGGGTTG
GTTTGCTGGTAGGAACCTGGCCATATCTCAGGTCACCACCAAATACGTTCTCTGGGTGG

ACGATGACTTTCTCTTCAATGACAAGACCAAGATTGAGGTGCTGGTGGATGTCCTAGA
GAAGACCGAACTGGATGTGGTAGGTGGCAGTGTGCTTGGAAATATGTTCCAGTTTAAG
CTGTTCCTGGAGCACAGTAAGAACGGGGACTGTCTCCATCGGCGGACAGGATCTTTCG
GGCCCCTGGACGGCTTCCCCAACTGCGTGGTGACCAGCGGCGTTGTTAACTTCTTCCT
GGCCCACACAGAGCGGCTCCAAAGAGTTGGCTTTGACCCCCGCCTGCAGCGAGTGGC
TCACTCAGAGTTCTTTATTGATGGGCTCGGGAGTCTGCTCGTGGGGTCCTGCTCAGATG
TGATTATAGGTCACCAGCCGCATTCTCCAGTGGAGGACCCAGAGCTGGCAGCCCTGGA
GAAGACCTACCGCATATACCGGGCCAACACCAATGATAAGATCCAGTTCAAGCTAGCTC
TCCACTACTTCAAGAACCACCTCCAATGTGCCACATAAAGATGCCTGGACTTCGGCAA
AGTGCTAGGTCTGGCTCAGTTACTGTAGGTCATTAGAAATGGACCAATCACTGATCAGA
ACAATGGCAACTATATTAATAATAATTATTGTTTGATGTCTTACCATTTGTGAATCACTGT
ACACACCTGATGTGAAGAGCTGACTCGTTTGAAAAGGCCCCAATGCTGGGAAAGATT
GAAGGCAGGAAGAGAAGGGGACGACAGAGGATGAGATGGTTAGATGGCACCACCAA
CTCAATGGACATGAGTTTGGGTAAACTGTGGAAGTCGGTGATGGACTGGGAGGCCTGG
TGTGCTGCGGTTCGTGGGCTCGCAAAGAGTTGGACACAACTGAGTGACTGAACTGAA
CTGAACTGTACATATAATGGTGGTGGTGGTGGTTTAGGCAATAAGTCGTGTCCAGCTTT
TGAATCACATTATCTCATGCAAAAGAGCACATGATGGATTGGTATCCTTGATCAAAAGG
ATCAAAAGGATTGGTATCCTTCTGGGTCCTTGAGGGTTATTTGGTGGGTATTACATCACA
GATCATCTCTAGCGTCAACTGTCATCCACAAATTTTTATTTGGTCATACTTTGTGGACTC
AAATAATGTGAATAACCCAGAGTCCTTACCTTGAGGAAGTGGCAGTCTGGTTCAGTTCA
TTCCACTAGTAGCATTGGGCAGCCAGGCTCAGAGGATCAGAGTGGGGAGAACTTACAA
GGGCCCATGCAGGTGAGGAGGGCTCTGTGGAGTCAGCTTACCTGCAGTCCAGCACATG
GATGGGATCCAGGAAAGTGGAGGGCAGAGATCACTGGGTAGTCCACTCACTGCTGGA
ATAAAGCTTTCCCTGGCATTAGATACTTACCATAAAGACACATTCCAGAATGCTTGGGAT
TGAACTTGGACCTTGTACTTTGTAAGCGCTGTGGCCAAGCCACTAAATTCCAGTTGCTT
ACTACTCAAGACTGCCATTGTCTGCAGAATGGAGCCATGGTGATGACGCGGGTTTTCTC
TGTCACCTTGGGAGTCAGTGGATTCTCCCACTGCTGCGCTGCCTTTTTGTTTCCACTCT
TTCCTACTGAAGACAGCCACGGTAGTTCAAAATCTCTGAAAAATGTGTGAGATGTTAAT
TCATGAAAAACGTTTTGGTTCTCAAATAATTTTTAAAAATACTGCATGCTCTATCTCCCT
CTTTGACAATCACAATTCATATTAGTATATTCAAGGCTCTGAGAAGTCCTGCTGTGAAG
AAATTGACTTAACGTCAATTCAGTATTTCTCAGATATGGATACAGACTGGCCATCATTTT
TTTTGTTCTCCTGAACACAAATAAAGCACATACCTCATTCCATTGTA
>B4GALNT2_Homo sapiens transcript variant 1, NM_153446.2
ATAGGTGGCTGCAGAGGCGAGGTGACGGCGCGTGCGGAACGAACTCTGCACCCCCAG
GAATGGGGAGCGCTGGCTTTTCCGTGGGAAAATTCCACGTGGAAGTGGCCTCTCGCGG
CCGGGAATGTGTCTCGGGGACGCCCGAGTGTGGGAATCGGCTCGGGAGTGCGGGCTT
CGGGGCTCTCTGCTTGGAACTCAGAGGCGCTGACCCAGCCTGGGGCCCGTTTGCTGCC
CACGGGAGGAGCCGCCGTCAGGGCTCGAGATTTCTGTGGCTCCTCAAGATATTGGTCA
TAATCCTGGTACTTGGCATTGTTGGATTTATGTTCGGAAGCATGTTCCTTCAAGCAGTGT
TCAGCAGCCCCAAGCCAGAACTCCCAAGTCCTGCCCCGGGTGTCCAGAAGCTGAAGC
TTCTGCCTGAGGAACGTCTCAGGAACCTCTTTTCCTACGATGGAATCTGGCTGTTCCCG
AAAAATCAGTGCAAATGTGAAGCCAACAAAGAGCAGGGAGGTTACAACTTTCAGGAT
GCCTATGGCCAGAGCGACCTCCCAGCGGTGAAAGCGAGGAGACAGGCTGAATTTGAA
CACTTTCAGAGGAGAGAAGGGCTGCCCCGCCCACTGCCCCTGCTGGTCCAGCCCAAC

CTCCCCTTTGGGTACCCAGTCCACGGAGTGGAGGTGATGCCCCTGCACACGGTTCCCA
TCCCAGGCCTCCAGTTTGAAGGACCCGATGCCCCCGTCTATGAGGTCACCCTGACAGC
TTCTCTGGGGACACTGAACACCCTTGCTGATGTCCCAGACAGTGTGGTGCAGGGCAGA
GGCCAGAAGCAGCTGATCATTTCTACCAGTGACCGGAAGCTGTTGAAGTTCATTCTTC
AGCACGTGACATACACCAGCACGGGGTACCAGCACCAGAAGGTAGACATAGTGAGTC
TGGAGTCCAGGTCCTCAGTGGCCAAGTTTCCAGTGACCATCCGCCATCCTGTCATACCC
AAGCTATACGACCCTGGACCAGAGAGGAAGCTCAGAAACCTGGTTACCATTGCTACCA
AGACTTTCCTCCGCCCCCACAAGCTCATGATCATGCTCCGGAGTATTCGAGAGTATTAC
CCAGACTTGACCGTAATAGTGGCTGATGACAGCCAGAAGCCCCTGGAAATTAAAGACA
ATCACGTGGAGTATTACACTATGCCCTTTGGGAAGGGTTGGTTTGCTGGTAGGAACCTG
GCCATATCTCAGGTCACCACCAAATACGTTCTCTGGGTGGACGATGATTTTCTCTTCAA
CGAGGAGACCAAGATTGAGGTGCTGGTGGATGTCCTGGAGAAAACAGAACTGGACGT
GGTAGGCGGCAGTGTGCTGGGAAATGTGTTCCAGTTTAAGTTGTTGCTGGAACAGAGT
GAGAATGGGGCCTGCCTTCACAAGAGGATGGGATTTTTCCAACCCCTGGATGGCTTCC
CCAGCTGCGTGGTGACCAGTGGCGTGGTCAACTTCTTCCTGGCCCACACGGAGCGACT
CCAAAGAGTTGGCTTTGATCCCCGCCTGCAACGAGTGGCTCACTCAGAATTCTTCATT
GATGGGCTAGGGACCCTACTCGTGGGGTCATGCCCAGAAGTGATTATAGGTCACCAGT
CTCGGTCTCCAGTGGTGGACTCAGAACTGGCTGCCCTAGAGAAGACCTACAATACATA
CCGGTCCAACACCCTCACCCGGGTCCAGTTCAAGCTGGCCCTCCACTACTTCAAGAAC
CATCTCCAATGTGCCGCATAAAGGTGTGAGGGCATAGGAGAAACACTAGGCTGGCTGG
TTATGGTATCTATAGCAGGCCACCAAAAACTGGACTCCTGATAGGTGAACGTTGTACCA
AACCAGCTGGTGGGTAGGGAAAAGGGAAATGGCTCAGTTACTGGAAGTACCAATCAA
AGGTGAAGGGTCACTGGAAATGAACCAGTCACTGACCAGGGCAATGGAGACTGTATT
AATAGCAATGATGATTTGTACAATGCCCTGCCTTTTTTGAAGCATTTGCAT
>B4GALNT2_Mus musculus, NM_008081.3
CTGCGGTAGGCAGTCTGCAGAAGTGGCTGGAGCGAGCTGCGATGACTTCCAGCGTGT
CTTTTGCCAGCTTCAGGTTTCCATGGCTCCTCAAGACATTTGTCCTCATGGTAGGACTT
GCCACTGTTGCGTTTATGGTGAGAAAGGTGTCCCTTACAACAGACTTCAGCACCTTCA
AGCCAAAGTTTCCAGAGCCTGCAAGGGTTGACCCAGTGCTGAAGCTTCTACCAGAGG
AGCATCTGAGGAAACTCTTTACCTACAGTGACATCTGGCTCTTCCCCAAAAATCAGTGT
GACTGTAACTCTGGCAAACTGCGAATGAAATATAAGTTTCAGGATGCCTATAACCAGAA
GGACCTTCCAGCTGTGAATGCCAGAAGACAGGCAGAATTTGAGCACTTTCAGAGGAG
AGAAGGGCTGCCTCGCCCACCACCTCTGCTGGCTCCACCCAATCTCCCCTTCGGATAC
CCAGTCCATGGTGTGGAGGTGATGCCCCTGCATACAATTCTCATCCCAGGCCTCCAGTA
TGAAGGGCCAGATGCTCCAGTCTATGAGGTCATCCTGAAAGCTTCTCTGGGGACACTG
AACACCCTTGCTGATGTGCCAGATGATGAGGTTCAGGGCAGAGGCCAGAGGCAGCTG
ACCATTTCCACCAGACATCGGAAGGTCCTGAATTTCATCCTTCAGCATGTGACGTACAC
CAGCACAGAGTACTATCTCCACAAGGTGGACACAGTAAGTATGGAATACGAGTCGTCA
GTGGCCAAGTTTCCAGTGACTATCAAACAACAGACTGTACCCAAGTTGTATGACCCTG
GACCTGAGAGGAAGATCAGAAACCTGGTGACCATTGCCACGAAGACTTTTCTCCGTCC
CCACAAGCTTAAGATCCTGCTTCAGAGTATTCGAAAATATTACCCAGACATCACCGTGA
TTGTAGCTGATGACAGCAAGGAGCCCCTGGAAATTAATGATGACTACGTGGAGTACTA
CACCATGCCCTTTGGGAAGGGCTGGTTTGCTGGGAGGAACCTGGCCATCTCACAGGTG
ACTACTAAATATGTCCTCTGGGTGGACGATGACTTTCTCTTCAGCGACAAGACCAAGAT

TGAGGTACTGGTGGATGTCCTGGAGAAAACCGAACTGGATGTGGTGGGTGGCAGCGT
GCAGGGGAATACTTACCAGTTCAGGCTGCTGTATGAACAGACCAAGAATGGGAGCTGT
CTTCACCAGAGGTGGGGATCATTCCAGGCCCTTGACGGCTTTCCCGGATGCACGTTGA
CCAGCGGCGTGGTGAACTTCTTCTTGGCTCACACGGAACAACTCCGAAGAGTTGGTTT
TGATCCCATCTTGCAACGAGTGGCCCACGGAGAGTTCTTTATTGATGGGCTGGGGAGA
CTGTTGGTGGGCTCTTGCCCGGGTGTAATTATAAACCACCAAGTAAGAACACCACCAA
AGGATCCAAAGCTGGCTGCCTTGGAGAAGACTTATGACAAATACCGGGCCAACACCAA
TTCTGTGATCCAATTCAAGGTTGCACTCCAGTACTTCAAGAACCATCTCTACTGCTCCA
CTTAAAAAGTACCAAGACCAGGAATCGCAATAAACAGATTAGCGGCGGGCAACAAAA
AAAATAGTAGGATTGGACATTGGAGGGTACCAAAAGCAGACTCCTAACAAGGGGAAC
AGGGAACAAAGAAGCTGGAAGCTGGAGCTAGGGTAGGACCCTGAAGTCGGCTCAGG
ACCCCCAAGACCAAATTCTCAGCACAGAGATTTATCTGCCCTAGAGAGAGAAAGGGCA
GGGAATGAGACAAAGACAAGGGCTGGAAGGTGGTGGGGCACACCTTTAATCACAGCA
CTCCGGGGGCAGAAGCAGGTGGATCTCTGCGCATTTTGAGGACAGCCTGGTCTACAGA
GAGAGTTCCAGGACAGCCAGGGCTACAGAGAAACTCTTATCTTAAAAAGGCAATAAGT
TGATCTAAAATAATGTTATTTAAAGCCAGATGATAAGGTTGGTGTAGTGTTGTGATCCCA
AAGCCCAGTAACTGAAGTAGGAGGATTGAGTTCAAGGCCAGCCTGGGCTATGTAACAG
CCCATCTTTAAATGAGTGACTAGGGGGCTGGAGAGAGCTCAGTGCTTAAGAGCGATTA
GGACAGAGGACCTAGATTGGCTCCCAGGACCCACATGATGAGTCACAACTTCATACCT
GCAGTTTCAGGGAATTGGAAGCATCTGCTTACCTCCAAAGGCTCCTGCATATGAGTGGT
GCATACATACACGCAGGCACACACATATGCACACAAAATACGCTTTAAAATAAATGAAG
GAATGAGTGAATGGCTAAATATTAACCAAATAATTCCGATTTTATTTTGTCAAGACTGCC
TGGCCTAGCACTCACTGTGTAAGACTGACCTCACACTCATGGCAATCCTCCTGTCTCCA
CTTCCCAGTGCTGAGGTATGAGGTGTGAACCACAATTTCTGGCTTGGATTTTTAAAAAG
AATTTACACACACACACATACATACACACACACTGTAGCTGTCTTCAGACACATCAGAA
AGGGCATCAGATCCCATTACAGATGGTTGTGAGCCACCATGTGGTTGCTGGGAATTGA
ACTCAGAACCTCTGGAAAAGCAGTCAGTGCTCTTAACTGATAAGCCATCTCTCTAGTCT
CTAGCTTGATTTTTAAACTTTGACTTTTAAATATACTTATTCATTATAATTTTTTTTCTTTT
TGAGACAGGGTTTCTCTGTGTAGCCCTGGCTGTCCTGGAACTCACTCTGTAGACCAGG
CTGGCCTCGAACTCAGAAATCCGCCTGCCTCTGCCTCCCGAGGGCTGGGATTAAAGGC
ATGCACCACCATGCCCGGCTTATATTTTGATTTTAATGGGATATTAAGATGGATGTTAAGA
TTGTGAGCTTTCAACCCTGTCTCGGAAAACCAAAAAAAAAAAAAAAAAAGAAAGAAA
AGATTGTGAGCCTTCATGTGGTTGTTGGGGATAGAATCTCCACTCACTCCAGTCAACCC
TGTTCTCAGTCCCTGCTAACTCCGGCCCAAAGATTTATTTATTATTATAATTATTATACGT
AAGTACACTGTAGCTGACTTCAGACACACTAGAAGAGTGTCAGATCTCATTACCGGTA
GTTGAGTCACCATGTAGTTGCGGGGACTTGAACTCAGGATCTTTGGAAGAGCAGTCCA
TCCTCTTACCCTCTGAGCCATCTCGCCAGCCCATTTCAACACTCTGAACATACTTACACT
TTATTTTTTACTTCCTGAGGTAAATATTGCTCTGAGCTAGACCATAGAAAGCCAAACAC
CAGTGGGTAAGGAGGCGAGGAATGACATGCCAGCTAGTTGTGGTGTGGGCCTTTAATC
CCAGCCTTGAATTCAGAGATCCTCTAGCCAGGGCTAGGCAGTGAGCTCTGTTTTGTTTT
GTTTTGTTCTGTTTTCAGAACAAAACCAAAATCAGGGGCTGAGGAAATGGCTCAAAAG
GTGAAGTGTTTGTTACCCAAGCATGAGGACTTGAGTTCAGATTCCCAGAAGTAAGGCA
TAGCTGCCAGTCTGTAACCCTGGGCATTCTCACAGCAAATGTTAACATCCTCCATTTG
ACTGGGTTGTCGTTAGCATGCTCAAACACAGTCTTTTCTCTACTTCAAGATCTAGAACC

ATCTCTCCAGAAGAGAGAGAGAGAGACAGAGAGTGAGTTAGTTTCCTATGATCCAGTA
ACACCTCTTCCCCAGGAACACAGGATGTTCTAAAAGGACATCCATTCAACTCAAAGGG
TTCAGGGACTCCCTGAAACCCACCAGACAGACTAGGCCCCCTCTTTTCCAAGTATATTA
GCTGTGAAGACTGCTGAGAGGCACTCTCAGACAAGTTGAGCTGCCTGAAACAGACAT
TCTCTAACCTGTTGAGCTGTCTGCAAGTTGTACAGAGGGCTTCAAGTTTACAGCTTTTG
TGACATGGTGAGCTTTCATGGTGCCGCTGTCTTTCGAGTCATTTGTGATCCTGCGGTAA
CCCCTCACCCATACTTCTGTCTGTAACCCCAATAAAACTCATCAGTCTGCTATACTGGA
>B4GALNT2_Rattus norvegicus, XM_008768250.2
ATGATCTACACACTAAATCCCCAACCCCGAGACCGAACTCTTATCTCCCCAGCTCTCGA
GATCAAAAATTCTAATGCCTTTTGCTTTTTCCCTGTGTCTTTTGCCAGCTCCAGGTTCCC
ATGGCTCCTCAAGACGTTAGTCATGATCCTAGGACTTGCCACTGTTGTGTTTATGTTCA
GAAGTTTGGCCCTTACAGCAGAGTTTGGCACCTTCAAGCCAGCGTTCCCAAAGCCTGG
GCCGGTTGACCCAGTGCTGAAACTTCTATCCGAGGAGCACCTGAGGACACTCTTTACC
TACAGTGACATCTGGCTCTTCCCCAAAAATCAATGCGACTGTGAGTCTGGCAAACAGC
AAATGAAATACAAGTATCAGAACGCCTATAACCAGAAGGACCTTCCGGCTGTGAACGC
CAGAAGACAGGCAGAATTTGAGCACTTCCAGAGGAGGGCAGGCCGGCAAAACGACT
TTCGTCTCCGTCTCCTTAAACGGAAAGGACAAACAGGGAGCTTGCGGTTCAGCTGGCA
TTCACTTCTACATTTTTCTCTAGAGAGGAAGCTCAGAAACCTGGTGACCATCGCCACG
AAGACTTTTCTCCGTCCCCACAAGTTTAAGATCCTGCTTCAAAGTATTCGAAAGTATTA
CCCAGACTTAACCGTGATTGTGACTGATGACAGCAAGGAGCCCCTGGAAATTAAAGAT
GACTACGTGGAGTATTACCCCATGCCCTTTGGGAAGGGCTGGTTTGCTGGTAGGAACC
TGGCCATCTCACAGGTGACTACTAAATACGTCCTCTGGGTGGACGATGACTTTCTCTTC
AGCAACAAGACCAAGATTGAGGTACTGGTGGATGTCCTGGAGAAAACTGAACTGGAT
GTGGTGGGCGGCAGCGTGCAGGGAAACACTTTCCAGTTCAGGCTGCTGTTGGAACGG
AGCGAGAATGGGGACTGTCTTCACCAGAGGTGGGGATCATTCCAGGCCCTCGATGGCT
TCCCCGGATGCACGCTGGCCAGTGGCGTCACGAACTTCTTCTTGGCTCACACAGAACA
GCTCCGAAGAGTCGGTTTTGATCCGGCCCTGCAAAGAGTTGCTCACCGAGAATTTTTA
TTGATGGGCTGGGGAGCCTGTTGGTGGGCTCGTGTCCGGGGGTGACTGTAGACCACCA
AACACGAACACCATCGAAGGATCCAAAGCTGGCTGCCTTGGAGATGTCTTACGCCAAA
TACCGGACCAACACCAATTCTCAGGTCCAGTTCAAGTTAGCGCTCCACTACTTCAAGA
ACCATCTCTACTGCGCCACTTAAAAGGCAAAGAGCCGGAAGTGCAATACGCAGATTAG
CTGTGGACAACAAAAAAATAGTAGGACTGGACATTAGGTGTGCTAAAAGCAGACTCCT
AACAAGGGGAACAGAGAGCGAAGTAGCTGGAAGTTGGAGCCAGTGTAGGGACCCTG
AAGTCGGCTCAAGATGCCCCAAAGACTAAGTTCTCAGAACAGAGATTTTCTGCCCCCG
AGGGGTAAAGGGCACAGAATAAGAGACAAAGACAGGGGCTGGCCAGTGGTGGTTTAC
ACCTTTAATTCCAGCACTCCGGAGGCAGAA
>B4GALNT2_Sus scrofa, NM_001244330.1
TCCGCGGAGTGGCACCACCAGCCCAGAATGTTCCGAGGCGGCGGGATTAAGGATGAC
TTCGTACAGCCCTAGATGTCTGTCGATCCTCAAGATATTGATGGTGCTTTTGGTCCTGAG
CGTTGGACTCTTTATGTTCCAAAGCGTGTTCCTCGATACAGACTTCAGTCTCCTCAACT
CACCCATCCCGTCCCCCACCCTGGATGCGCAGACGCTGAAGCTTCTACCTGAGAAACC
CGATTTCTACGGTGAAAACGGGCTGTTCCCGAAAAACCAGTGCCAATGTGACGCCTTC
GGGCATCAGGAAAGCTATAACTTGGAGGATGCCTACGACCCGCAAGACCTCCCCGCAG
TGAACCTGAGGAGACAGGCTGAGCTCGAACACTTTCAGAGGAGAGAAGGGCTCCCTC

GCCCACCGCCCCTGCTGGCTCAGCCCAACCTCCCCTTTGGGTACCCGGTCCACGGGGT
GGAAGTGATGCCTCTACACACCATCCCCATCCCAGGCCTCCGGTTTGAAGGACCTGAT
GCTCCCATCTATGAGGTCACCCTGACAGCTTCTCTGGGGACACTGAACACCCTTGCTG
ACGTCCCAGACAATGTGGTGAAGGGCAGAGGCCAGAAGCAGCTGAACATTTTGACCA
GTAGCCGGGAGCTTTTGAATTTCATCCTCCAGCATGTGACATACACGAGCACAGAGTAC
CACCTCCACAGAGTGGATGTGGTGAGTCTGGAGTCCAAGTCCTCAGTGGCCAAGTTTC
CAGTGACCATCCGCTATCCTGTCATGCCCAAGTTATATGACCCTGGACCAGAGAGGAAG
CTCCGAGACCTGGTGACCATTGCCACCAAAACCTTCCTCCGTCCCCACAAGCTCATGA
CCATGCTCCGGAGTGTTCGTGAGTACTACCCAGACCTGACGGTGATCGTGGCCGATGA
CAGCAAGGAGCCCCTGAAAATCACTGACAGCCACGTGGAGTATTACACCATGCCATTT
GGGAAGGGCTGGTTTGCTGGCAGGAACCTGGCCATATCTCAGGTCACCACCAAATATG
TGCTCTGGGTGGACGATGACTTCATCTTCAACAGCAAGACCAGGATCGAGGCGCTGGT
GGACGTCCTAGAGAAAACGGAACTGGACGTGGTAGGTGGCAGCGTGATTGAAAACAC
ATTCCAGTTCAAGCTGTTGCTGGAGCAGGGGAAGAATGGCGACTGTCTCCACCAGCA
GCCAGGATTTTTCCGGCCCGTGGATGGCTTCCCCGACTGCGTGGTGACCAGTGGTGTT
GTCAACTTCTTCCTGGCTCACACAGAGCGACTCCAAAGAATTGGCTTCGACCCCCGGC
TGCAGCGAGTGGCTCACTCAGAGTTCTTTATTGATGGGCTCGGGAGCCTGCTCGTGGG
GTCCTGCCCACACGTGATCATAGGTCACCAGCCCCATTTACCAGTGATGGACCCAGAG
CTGGCCACCCTGGAGGGGAACTACACCAGTTATCGGGCCAACACCGAAGCCCAGATC
AAATTCAAGTTGGCTCTCCACTACTTCAAGAACTATCTCCAATGTGTCACCTAAGGTAT
CCGGGCATTGGAAAAGCGCTGAGCTGCCTGGTTGCAAGTATCTAAGACAGCGGATGCG
GTGGCTGGGATACCAATATTTGAACTCCTCATAAGATAAGCACTGTAATGCCCAGGGAG
CAGGGTAGGCAGGTGGGTCTGACTCCGTTACTGGAAGTACCAATAAAAGTACAGGGTC
ATTAGAAATGGACCAGTCACTGAGGTGGGCAATGGAGACTTCATTCATAACGATTACG
GCGGTGTTTCCATCATGGCTCAGAGGTAGCAATCCAGACTGCTATCCACGAAGATGCG
AGTTGGATCCCTGGCCTTGCTCAGTGGGCTAAGGATCTGGCATTGCTGTGGCTGTGGCA
TAGGCTGGCAGCTGCAGCTCTGATGCGCCCCCTAGCCTGGGAACTTCCAGATGCTAAG
TGTGTGGCCATAAAAAAAAAAAAAAAAAAAAAAAAAAA
>B4GALNT2_Xenopus tropicalis, XM_002935708.4
ATATGTTTATTGTACTGAGCAATTCATTCTCCCAGGCTCTGGCTCTGATTATCCTCTCCTG
CACTGCGAGTGGATTTCATACAAACTGACAAACATGTTCCTGAAGGGAAAACGCACTT
GTTCAGGGTTTGGGCCCCGGATATGGCTGTTTGTTATTTTTATTGTGATTGTAGCAGGTT
TCGCTTTTCTGCAGCACAGGAAAAATGGGCTTATTGGCTTGACCGCCATTCATCACCCC
AAGAGACAACTGCCACCTTTGTATTCTGATGAAGAACTTATGTCCAAGTTCATCTACAA
TGATGTCAACCTCTACCAAAATAGACAATGTTTTTGCCCCACTGAGCCACAGAGGATTG
AAGTTTATCATCTTAAGAAAAATCTGAATCCAAATGATGCAGACGCTATGAAAGCCAGA
AGAAAGAAGGAGTATGAGCATTACAGCAAGAGGCAACATTCCCATGATATTCTCATAG
CTACTCCAAACTCCCCTCTTGGCTATCCAGCCCATGGTATCCAGGTTAAGCCTCTCCATA
CTATCCAGCTACCAGGGTTACAAGTAAATGCCGATCTTCAGAATTATGCGGTAACATTG
GAAGCTTCCTTGGGGACTTTTGACACCCTAGTGAATACATTGGATATAAGCAACAATGT
AGTTTTTGGTAGAGGAGAAAAACAGTTTGTTATAAGGACATCTGACCAGAATTTACTAA
ACCACATCCTGCGCCACATCACGTACACGAGTACAGTTTACGATATCGACAGTTTGGAT
ATCATTAAATTTACTATGGATCAACATGAAGCTAAGATTCCAGTATCGATCCGGCAGCCA
CCAATGATACATTTATATGACCCAGGGGCAGAGCAGAAGATTGAGTCCTTGGTTACAAT

AACCACCAAGACTTTCCTGCGCTATGACAAGCTGAAAACTCTCATAAGTAGCATTCGC
CAGTACTACCCGGACATCAAGATCATTGTAGCAGATGACAATAACGTCACTGAGAAGA
TCGAGGATAAGAACGTTGAGCAGTATTTCATGCCGTTTGGAAAGGGATGGTTCGCTGG
AAGGAATCTTGCCGTATCTCAGGTGACCACCAAATATTTCCTTTGGGTGGATGACGATT
TCCTTTTCACTGCAGACACCAAGATAGAAAAGTTGGTAAATGTGTTGGAGTCAACCAA
CTTAGATGTGGTTGGGGGCAATGTGAATGGCAATCATTTTAGCTTTAGACTCCTGCTAC
AAGATGGCGGTGAGGAGGGAGACTGTCTGCACTGGAGAGGAGGGAGCTACCGAGGTA
TTGAAGGCTTTCCAAAATGTGTGCTAACCGGAGGAGTTGTCAACTTTTTCTTGGCGCA
CACAGACCGCGTCTTAAGTGTTGGGTTTGACCCAAAGCTTCAGAGAGTGGCACATACT
GAGTTTTTTGTTGATGCGCTGGGCCGACTACGCGTAGGATCTTGCAGTGACGTCGTTGT
TGGACACCAAAAAAAGGACCAATCAGTTTCTGGAGCCATGGCTGAACAGGCCAAAAA
ATATAATTCTTTTAGAACAGATACTAAAGAACAAGTGAAAATGAAACTCACACTTTATTA
TTTCAAGAACCGATTAAGCTGCTTTACAAAACATTAAAACTGTTGGAATCAGGAATGG
AAATATATTATCTCAGGGGGCGGATGTTCAAGCCTTAGGTGAATAGATGTAGGGGTTGT
CCGTCACTGTGGTTCAAGTTAATTAATGTGTTTCAGGTTCTAAGAGAGATTTCATGGTAT
ACTTACCAAATGTGATTCCGAGCTATAATACATAGAACAGCTAGGATGGACTGAGTGTT
GGGAAGAAAGTCACTCTGTTCAAGGAACACTGGATAATGTATAAAAGAATTTATCTTCC
TTTTTGTGCATGGAGAATGGACATTTCCCTGTGAATTTTACCTGGCGGCAGGTATTATTA
TACATAACAGAAAAAAAGTGCTTCTTTACCATGGTGTCAGACATTAACAGGGCAATATA
TCGGCCACCAATGGGAAGATGTCTTGCCACATGGAACCTGTTGAGTCAACAGGCAACT
GAACTCTGTTAAGTCGAGCAATAAAGCAACTCCAGCTCATAGATGAATACAAATAGGC
AGAAGGAATATTCTGTGCACACAAGATATACTAATTAATAATGCTATTAATGTATTTTTAT
TGTTGGAGAAAAATCTAAATCATAAATGTAAGGGCATCAATATATTTCTACATGTAATTA
ATAGATAATTTATATCCTATAATGTGACCTATCAAAGAGTCAAATTGACGTACATGTCAAT
AAATATTTTTGTTATCACTTTGA
>B4GALNT1_Ovis aries transcript variant X1, XM_012176064.1
ATGTGGGAGAGGGCGAGAAGAAAGGAGGCCAGGAGACCCGAGGCCCGGGGAGTGAG
GGGAGGGGGACGAGGGTACCTGGGGCTCCTGTACGCGAGCACCCGGGGCGCGCCGG
GCCTCCGGGCACCTCTTGCGCTGTGGACGCCCCTACAGGGCAACCCCAGGCCGGAAC
TGCTAGGTCTTGCCCCTGAGCCCAGATACGCACACATCCCGGTTAGGATCAAGGAGCA
AGTGGTGGGGCTGCTGTCTGCGAACAATTGCAGTTGTGAGTCCAGTGAGGGACGCCT
CCACCTCCCCTTCCAGAGGCAGGTCCAAGCCTTTGACTTCACCAAGGCCTTTGACCCT
GAGGAGCTGAGCACTGTGTCTGCCTCGAGGGAGCAGGAGTTCCAGGCCTTCCTTTCA
AGGAGCCAGTCTGCTGCTGACCAGCTGCTCATAGCCCCTGCCAACTCCCCGCTACAGT
ACCCCCTGCAGGGTGTGGAGGTCCAGCCCCTCAGAAGTATCTTGGTACCAGGACTGGG
CCTGCAAGCCACTTCTGGTCAGGAGGTATACCAGGTGAACCTGACTGCCTCCTTGGGC
ACCTGGGACGTGGCAGGGGAAGTAACTGGAGTGACTCTCACCGGAGAAGGGCAGCC
AGATCTCACCCTCACCAGCCCAGGGCTGGACCAACTCAATCGGCAGCTGCAACTGGTC
ACTTATAGCAGCCGAAGCTACCAGGCAAATACAGCAGACACAGTCCGGTTCGCCACTG
AGGGACACGAAGCTGCCTTCACCATCCGCATAAGACACCCACCCAACCCTCGGCTGTA
CCCACCTGGGTCTGTACCCCAGGGAGCCCAGTACAACATCAGCGCTCTGGTCACCATT
GCCACTAAGACCTTCCTTCGTTACAATCGGCTACGGGCACTCATCGCCAGCATCCGCCG
CTTCTACCCAACGGTCACAGTGGTAATCGCCGATGACAGCGACAAGCCAGAGAGCGTT
AGAGGTCCCCACATCGAGCACTATCTCATGCCTTTTGGCAAGGGCTGGTTCGCAGGCC

GGAACCTGGCCATATCCCAAGTAACCACCAAGTACGTGCTGTGGGTGGACGATGACTT
CGTCTTCACGGCGCGGACTCGACTAGAGAGGCTTGTGGACGTGTTGGAGCGGACGCC
GCTGGACCTGGTGGGGGGCGCGGTGCGCGAAATCTCCGGCTTCGCCACCACCTACCG
GCAGGCGCTGAGCGTGGAGCCTGGTGCGCCAGGCCGGGGGAACTGCCTCCGGCAGA
AGCGCGGCTTCCACCACGAGCTCGTCGGCTTCCCGGGGTGCGTGGTCACCGACGGCG
TGGTCAACTTCTTCCTGGCGCGCACTGACAAGGTGCGCGAGGTCGGCTTTGACCCGCG
CCTCAGCCGCGTGGCGCACCTGGAATTCTTCCTGGATGGACTTGGTTCTCTTCAAGTG
GGCTCCTGCTCAGACGTGGTTGTGGATCACGCATCCAAGTTGAAGTTGCCTTGGACCT
CAAGGGATGCGAAGGCAGAGACTTATGCTCGGTACCGTTACCCGGGATCACTGGACGA
GAGTCAGGTGGCCAAACATCGTCTGCTCTTCTTCAAACACCGGCTCCAGTGCATGACC
TCAGAGTGATGGCCACTTGGGGCCTTTCAGTTGTCAGACTGGGCCTGCCTCCTTGTCC
CTGCCAGGAATTCCTAGCAAACCCCACCAGCCAGTGACCACTCCACTGACTGTCCCAG
TCTCCTTCAGAACTTGATCCCAAACAGGGGCTTGTCCTGGTGTCACTCCTCCTTTCTGC
AAGTGCCCAGGGGCATGATGGAGCCATAACTTGTCCCACAGCCAGTGCCAAGTCCTCC
CCCCAACCCCTTCCCCTAGGGCAGGAAGTGGGGGGTTGCTTTTCAAGTGCCAAAGAG
CCCATAGGGGGACTCTAAAAACCTAAAGCAGAAATACTCTCATCTCCTTGGTACTAAGG
GGTTGGGGAAGCCCCCAGTATATAGTCCCAGGGCTGTGCCCCTATCTCTGGATCCAGGA
CTCTGTGCAGTGGCTGCAGCCTCACCCCCATGGAGAGAATGGAGTTGGAATGGGGGCT
GGTGAACATACAGGGGAAAGCCGTTTTTAGCTGTGTCTTTGCAGTGCTTTGGGAGTGG
AGGAAGGGGCAC
>B4GALNT1_Ovis aries transcript variant X2, XM_012159415.2
ACCTGCATCTCTTAACCTCTCCCGCCTCTGCAGGCAGGTTCTTTACCACTAGTGCCACC
TGGGAAGCTCCTCAATTTCCCTACTTTCCTCACTTTTCTTTCTCAAAGGACGTGAGCCT
TCTACTAATATGCTCTTCAATATCTTTCAGTCTTTCACTAACACTCCTAGATTCCTTCCTG
GTTCTACCCAAGACCCAGTTCCAAATCCATTCCCACATTTTTAGGTTTTTATTATGGCAG
CACTTCATTTCTAGTATTTGCACAATCCTAGAAGCTAAAACCTACACTCAGGAGCTGAC
TATATATGCTATTGTTTATTTTGCTTAACAGCTTTGGATATCATTCAGTATCAGTAGATATA
GATTAATCCAGTGTCCCATTGTCTGACTGAATCATATTTAAAAATTTTAACTTTCTAATAT
TTAAGATTTAAATGATTAATATTTTATTTTTTGATTAATATTTTAATTGTTAATAGATTGGC
CAATTGCTCTAAGCTGGGTCCATTAACACTCACAACAACGTATGGTAGTGACTATTTCC
TCAAATACTTGATCACTTGTGTTAGCAAACTCTTTGACCTTTACTAAATTGCTAAACAA
AAACCATTTCATTGTTACTCTAATTTGGATTTTACTTATTATGAATAGGGTCAAGTGTACA
TTCATTTGTTTGAAAAGCCATTTTTACTTTTTCCCTCAACTGTCTAATCACAATTTTTGC
CCATTTGGGGGTGACTGATAATTTTTTCCTATTGATGTTTTATAGCTCTTAAGAAAAATA
GGCCTTGATCTGTCTTACATGTTGCACATATTTTTTCACAGTTTGTCATTTCTCAAGTTT
GTTTATAGAAATTTCTTCACCCAAGCTTATTATATAGGCAAATCTATTAATACGTCCTTTT
ATCTTACTTAGAGATATCTTCCCCACTCCAAGATCATAAAAAAGAACAAAACAAACTCC
ATGAATTATTTGACTACTCTTTGTGGTTACATTTTTAATAATAAGATATTTGATACATCTG
GAATTTATTTTGGTGTAAAGAGGGTGAGAATCTCACTTTCTCTCCAGCCGATTAGTCATT
TCTTTGTACACAGTTCATCCAATAATCCAACCGTCCCTCTCCCATGCCCCCACATTTTCA
TTATGTTTTACATTTCCTATTATATCTTTTTTCCCTATTAAATTGTGACATCTTTTATTAGAA
CCATGGGACCACAGGACATAGGGCAGCAGCAAGAATCCAGCTACAGCTAAGGTAGTAC
AGGTCTGCAGCTTGCACGCCTAAGTACAACCAATCTAGGCACTGTGTGGGGTTTCTTTC
CTTCCGCAAGTCTGCAACAAGGAGCAGTCAGAAGAAGCAGGCCCTTGAATTACTGAG

TGTTTGTTACATCATTGAGTATTCGTTAGATAACTGGGGTATGCGGGGGCTGGAGGGCG
GGAGAGGCGAGGCTAGGAGGGAGGCTGTCAACTTGTCTTTGTTAGCTAGGCAGTCTTT
ATTTTCCAGTCTTGTCTGGGTTCCTTTATCTCACCCTAAGTATCCAAATCTTCCTTGAAA
TGGGGTTACAGGGCGTCCCCACCCCATCACTTGCCAAACCCTTATCGCCTGTCCTGGCC
TACAGTCTCGATGGAAAGACGGAAAATAGGAAAAGAGTGATTGGATCGCGGTGGGGC
GGGGGAGACGGCCAGGGTTCGGAGGCAAAGGGCTGGCGGGGAGGGAGGAGGCGTG
AAGAGGACCTTAGTGCTTCGCTGGCTTCGCGCCCCCGCAGTGTCCTCCGCGCCCACCA
GAAGGCGCCCCAGCGCTCTCATTCTCGGCGCGTAGCCTGACGCTCCTCCGCAACCGGC
ACCCGAGCCGAGCCGCCGGGATCTGAAAAGCCGGGCGGCGGCCGGGACTTTCCCGGC
CCGGCAAGTGGGACAGGGTCCCAGGGAACCCCGGATTTGTTTCTGGGATTGGCGTGC
GGCGGTTTCTCGACTCCGATCCCCCAACCCCGCAGGAAACGCCCAGAACCACCCCAG
CGCGGTGGTCTACAAAGCCCGGAGAGTCCAAAGGCGGGCGGGGTCGTGCGCGCTCGG
GAGGCAGGAGGACTCCCCCTCACTCAGATTCACGCCCGGCTCCCCAATCAGGACGCCT
CCAGCCCCATGAGGACTCTCAGGCCAGGGCTGAGAGCCTGGCACAGCCCTGACCGAA
ATCTTGCAGCGGCCTTAAAGCGTTAAGACAGGATGCGGCTGGGCCGCCGGGCCCTCGG
TGTGCTCGTCCTGCTGCTCGCCTGCGCCTCGCTGGGGCTCCTGTACGCGAGCACCCGG
GGCGCGCCGGGCCTCCGGGCACCTCTTGCGCTGTGGACGCCCCTACAGGGCAACCCC
AGGCCGGAACTGCTAGGTCTTGCCCCTGAGCCCAGATACGCACACATCCCGGTTAGGA
TCAAGGAGCAAGTGGTGGGGCTGCTGTCTGCGAACAATTGCAGTTGTGAGTCCAGTG
AGGGACGCCTCCACCTCCCCTTCCAGAGGCAGGTCCAAGCCTTTGACTTCACCAAGGC
CTTTGACCCTGAGGAGCTGAGCACTGTGTCTGCCTCGAGGGAGCAGGAGTTCCAGGC
CTTCCTTTCAAGGAGCCAGTCTGCTGCTGACCAGCTGCTCATAGCCCCTGCCAACTCCC
CGCTACAGTACCCCCTGCAGGGTGTGGAGGTCCAGCCCCTCAGAAGTATCTTGGTACC
AGGACTGGGCCTGCAAGCCACTTCTGGTCAGGAGGTATACCAGGTGAACCTGACTGCC
TCCTTGGGCACCTGGGACGTGGCAGGGGAAGTAACTGGAGTGACTCTCACCGGAGAA
GGGCAGCCAGATCTCACCCTCACCAGCCCAGGGCTGGACCAACTCAATCGGCAGCTG
CAACTGGTCACTTATAGCAGCCGAAGCTACCAGGCAAATACAGCAGACACAGTCCGGT
TCGCCACTGAGGGACACGAAGCTGCCTTCACCATCCGCATAAGACACCCACCCAACCC
TCGGCTGTACCCACCTGGGTCTGTACCCCAGGGAGGTGAGACTGCCCAGTACAACATC
AGCGCTCTGGTCACCATTGCCACTAAGACCTTCCTTCGTTACAATCGGCTACGGGCACT
CATCGCCAGCATCCGCCGCTTCTACCCAACGGTCACAGTGGTAATCGCCGATGACAGC
GACAAGCCAGAGAGCGTTAGAGGTCCCCACATCGAGCACTATCTCATGCCTTTTGGCA
AGGGCTGGTTCGCAGGCCGGAACCTGGCCATATCCCAAGTAACCACCAAGTACGTGCT
GTGGGTGGACGATGACTTCGTCTTCACGGCGCGGACTCGACTAGAGAGGCTTGTGGAC
GTGTTGGAGCGGACGCCGCTGGACCTGGTGGGGGGCGCGGTGCGCGAAATCTCCGGC
TTCGCCACCACCTACCGGCAGCTGCTGAGCGTGGAGCCTGGTGCGCCAGGCCGGGGG
AACTGCCTCCGGCAGAAGCGCGGCTTCCACCACGAGCTCGTCGGCTTCCCGGGGTGC
GTGGTCACCGACGGCGTGGTCAACTTCTTCCTGGCGCGCACTGACAAGGTGCGCGAG
GTTGGCTTTGACCCGCGCCTCAGCCGCGTGGCGCACCTGGAATTCTTCCTGGATGGAC
TTGGTTCTCTTCAAGTGGGCTCCTGCTCAGACGTGGTTGTGGATCACGCATCCAAGTTG
AAGTTGCCTTGGACCTCAAGGGATGCGAAGGCAGAGACTTATGCTCGGTACCGTTACC
CGGGATCACTGGACGAGAGTCAGGTGGCCAAACATCGTCTGCTCTTCTTCAAACACCG
GCTCCAGTGCATGACCTCAGAGTGATGGCCACTTGGGGCCTTTCAGTTGTCAGACTGG
GCCTGCCTCCTTGTCCCTGCCAGGAATTCCTAGCAAACCCCACCAGCCAGTGACCACT

CCACTGACTGTCCCAGTCTCCTTCAGAACTTGATCCCAAACAGGGGCTTGTCCTGGTG
TCACTCCTCCTTTCTGCAAGTGCCCAGGGGCATGATGGAGCCATAACTTGTCCCACAGC
CAGTGCCAAGTCCTCCCCCCAACCCCTTCCCCTAGGGCAGGAAGTGGGGGGTTGCTTT
TCAAGTGCCAAAGAGCCCATAGGGGGACTCTAAAAACCTAAAGCAGAAATACTCTCCT
CTCATCTCCTTGGTACTAAGGGGTTGGGGAAGCCCCCAGTATATAGTCCCAGGGCTGTG
CCCCTATCTCTGGATCCAGGACTCTGTGCAGTGGCTGCAGCCTCACCCCCATGGAGAG
AATGGAGTTGGAATGGGGGCTGGTGAACATACAGGGGAAAGCCGTT
>B4GALNT1_Ovis aries transcript variant X3, XM_012159413.2
ACCTGCATCTCTTAACCTCTCCCGCCTCTGCAGGCAGGTTCTTTACCACTAGTGCCACC
TGGGAAGCTCCTCAATTTCCCTACTTTCCTCACTTTTCTTTCTCAAAGGACGTGAGCCT
TCTACTAATATGCTCTTCAATATCTTTCAGTCTTTCACTAACACTCCTAGATTCCTTCCTG
GTTCTACCCAAGACCCAGTTCCAAATCCATTCCCACATTTTTAGGTTTTTATTATGGCAG
CACTTCATTTCTAGTATTTGCACAATCCTAGAAGCTAAAACCTACACTCAGGAGCTGAC
TATATATGCTATTGTTTATTTTGCTTAACAGCTTTGGATATCATTCAGTATCAGTAGATATA
GATTAATCCAGTGTCCCATTGTCTGACTGAATCATATTTAAAAATTTTAACTTTCTAATAT
TTAAGATTTAAATGATTAATATTTTATTTTTTGATTAATATTTTAATTGTTAATAGATTGGC
CAATTGCTCTAAGCTGGGTCCATTAACACTCACAACAACGTATGGTAGTGACTATTTCC
TCAAATACTTGATCACTTGTGTTAGCAAACTCTTTGACCTTTACTAAATTGCTAAACAA
AAACCATTTCATTGTTACTCTAATTTGGATTTTACTTATTATGAATAGGGTCAAGTGTACA
TTCATTTGTTTGAAAAGCCATTTTTACTTTTTCCCTCAACTGTCTAATCACAATTTTTGC
CCATTTGGGGGTGACTGATAATTTTTTCCTATTGATGTTTTATAGCTCTTAAGAAAAATA
GGCCTTGATCTGTCTTACATGTTGCACATATTTTTTCACAGTTTGTCATTTCTCAAGTTT
GTTTATAGAAATTTCTTCACCCAAGCTTATTATATAGGCAAATCTATTAATACGTCCTTTT
ATCTTACTTAGAGATATCTTCCCCACTCCAAGATCATAAAAAAGAACAAAACAAACTCC
ATGAATTATTTGACTACTCTTTGTGGTTACATTTTTAATAATAAGATATTTGATACATCTG
GAATTTATTTTGGTGTAAAGAGGGTGAGAATCTCACTTTCTCTCCAGCCGATTAGTCATT
TCTTTGTACACAGTTCATCCAATAATCCAACCGTCCCTCTCCCATGCCCCCACATTTTCA
TTATGTTTTACATTTCCTATTATATCTTTTTTCCCTATTAAATTGTGACATCTTTTATTAGAA
CCATGGGACCACAGGACATAGGGCAGCAGCAAGAATCCAGCTACAGCTAAGGTAGTAC
AGGTCTGCAGCTTGCACGCCTAAGTACAACCAATCTAGGCACTGTGTGGGGTTTCTTTC
CTTCCGCAAGTCTGCAACAAGGAGCAGTCAGAAGAAGCAGGCCCTTGAATTACTGAG
TGTTTGTTACATCATTGAGTATTCGTTAGATAACTGGGGTATGCGGGGGCTGGAGGGCG
GGAGAGGCGAGGCTAGGAGGGAGGCTGTCAACTTGTCTTTGTTAGCTAGGCAGTCTTT
ATTTTCCAGTCTTGTCTGGGTTCCTTTATCTCACCCTAAGTATCCAAATCTTCCTTGAAA
TGGGGTTACAGGGCGTCCCCACCCCATCACTTGCCAAACCCTTATCGCCTGTCCTGGCC
TACAGTCTCGATGGAAAGACGGAAAATAGGAAAAGAGTGATTGGATCGCGGTGGGGC
GGGGGAGACGGCCAGGGTTCGGAGGCAAAGGGCTGGCGGGGAGGGAGGAGGCGTG
AAGAGGACCTTAGTGCTTCGCTGGCTTCGCGCCCCCGCAGTGTCCTCCGCGCCCACCA
GAAGGCGCCCCAGCGCTCTCATTCTCGGCGCGTAGCCTGACGCTCCTCCGCAACCGGC
ACCCGAGCCGAGCCGCCGGGATCTGAAAAGCCGGGCGGCGGCCGGGACTTTCCCGGC
CCGGCAAGTGGGACAGGGTCCCAGGGAACCCCGGATTTGTTTCTGGGATTGGCGTGC
GGCGGTTTCTCGACTCCGATCCCCCAACCCCGCAGGAAACGCCCAGAACCACCCCAG
CGCGGTGGTCTACAAAGCCCGGAGAGTCCAAAGGCGGGCGGGGTCGTGCGCGCTCGG
GAGGCAGGAGGACTCCCCCTCACTCAGATTCACGCCCGGCTCCCCAATCAGGACGCCT

CCAGCCCCATGAGGACTCTCAGGCCAGGGCTGAGAGCCTGGCACAGCCCTGACCGAA
ATCTTGCAGCGGCCTTAAAGCGTTAAGACAGGATGCGGCTGGGCCGCCGGGCCCTCGG
TGTGCTCGTCCTGCTGCTCGCCTGCGCCTCGCTGGGGCTCCTGTACGCGAGCACCCGG
GGCGCGCCGGGCCTCCGGGCACCTCTTGCGCTGTGGACGCCCCTACAGGGCAACCCC
AGGCCGGAACTGCTAGGTCTTGCCCCTGAGCCCAGATACGCACACATCCCGGTTAGGA
TCAAGGAGCAAGTGGTGGGGCTGCTGTCTGCGAACAATTGCAGTTGTGAGTCCAGTG
AGGGACGCCTCCACCTCCCCTTCCAGAGGCAGGTCCAAGCCTTTGACTTCACCAAGGC
CTTTGACCCTGAGGAGCTGAGCACTGTGTCTGCCTCGAGGGAGCAGGAGTTCCAGGC
CTTCCTTTCAAGGAGCCAGTCTGCTGCTGACCAGCTGCTCATAGCCCCTGCCAACTCCC
CGCTACAGTACCCCCTGCAGGGTGTGGAGGTCCAGCCCCTCAGAAGTATCTTGGTACC
AGGACTGGGCCTGCAAGCCACTTCTGGTCAGGAGGTATACCAGGTGAACCTGACTGCC
TCCTTGGGCACCTGGGACGTGGCAGGGGAAGTAACTGGAGTGACTCTCACCGGAGAA
GGGCAGCCAGATCTCACCCTCACCAGCCCAGGGCTGGACCAACTCAATCGGCAGCTG
CAACTGGTCACTTATAGCAGCCGAAGCTACCAGGCAAATACAGCAGACACAGTCCGGT
TCGCCACTGAGGGACACGAAGCTGCCTTCACCATCCGCATAAGACACCCACCCAACCC
TCGGCTGTACCCACCTGGGTCTGTACCCCAGGGAGCCCAGTACAACATCAGCGCTCTG
GTCACCATTGCCACTAAGACCTTCCTTCGTTACAATCGGCTACGGGCACTCATCGCCAG
CATCCGCCGCTTCTACCCAACGGTCACAGTGGTAATCGCCGATGACAGCGACAAGCCA
GAGAGCGTTAGAGGTCCCCACATCGAGCACTATCTCATGCCTTTTGGCAAGGGCTGGT
TCGCAGGCCGGAACCTGGCCATATCCCAAGTAACCACCAAGTACGTGCTGTGGGTGGA
CGATGACTTCGTCTTCACGGCGCGGACTCGACTAGAGAGGCTTGTGGACGTGTTGGAG
CGGACGCCGCTGGACCTGGTGGGGGGCGCGGTGCGCGAAATCTCCGGCTTCGCCACC
ACCTACCGGCAGCTGCTGAGCGTGGAGCCTGGTGCGCCAGGCCGGGGGAACTGCCTC
CGGCAGAAGCGCGGCTTCCACCACGAGCTCGTCGGCTTCCCGGGGTGCGTGGTCACC
GACGGCGTGGTCAACTTCTTCCTGGCGCGCACTGACAAGGTGCGCGAGGTTGGCTTTG
ACCCGCGCCTCAGCCGCGTGGCGCACCTGGAATTCTTCCTGGATGGACTTGGTTCTCTT
CAAGTGGGCTCCTGCTCAGACGTGGTTGTGGATCACGCATCCAAGTTGAAGTTGCCTT
GGACCTCAAGGGATGCGAAGGCAGAGACTTATGCTCGGTACCGTTACCCGGGATCACT
GGACGAGAGTCAGGTGGCCAAACATCGTCTGCTCTTCTTCAAACACCGGCTCCAGTGC
ATGACCTCAGAGTGATGGCCACTTGGGGCCTTTCAGTTGTCAGACTGGGCCTGCCTCC
TTGTCCCTGCCAGGAATTCCTAGCAAACCCCACCAGCCAGTGACCACTCCACTGACTG
TCCCAGTCTCCTTCAGAACTTGATCCCAAACAGGGGCTTGTCCTGGTGTCACTCCTCCT
TTCTGCAAGTGCCCAGGGGCATGATGGAGCCATAACTTGTCCCACAGCCAGTGCCAAG
TCCTCCCCCCAACCCCTTCCCCTAGGGCAGGAAGTGGGGGGTTGCTTTTCAAGTGCCA
AAGAGCCCATAGGGGGACTCTAAAAACCTAAAGCAGAAATACTCTCCTCTCATCTCCT
TGGTACTAAGGGGTTGGGGAAGCCCCCAGTATATAGTCCCAGGGCTGTGCCCCTATCTC
TGGATCCAGGACTCTGTGCAGTGGCTGCAGCCTCACCCCCATGGAGAGAATGGAGTTG
GAATGGGGGCTGGTGAACATACAGGGGAAAGCCGTT
