# Supplementary figures and images for: Molecular Cloning of the B4GALNT2 Gene and Its Single Nucleotide Polymorphisms Association with Litter Size in Small Tail Han Sheep
Source: Animals (Basel). 2018 Sep 20;8(10):160. doi: 10.3390/ani8100160 (PMC6210199; doi:10.3390/ani8100160)

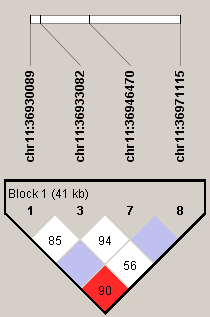

Supplement: Supplementary file 1 [file animals-08-00160-s001.zip › Supplementary file/Supplementary file 2.png]

Supplementary file 3: Predicted secondary structures of ovine B4GALNT2 protein

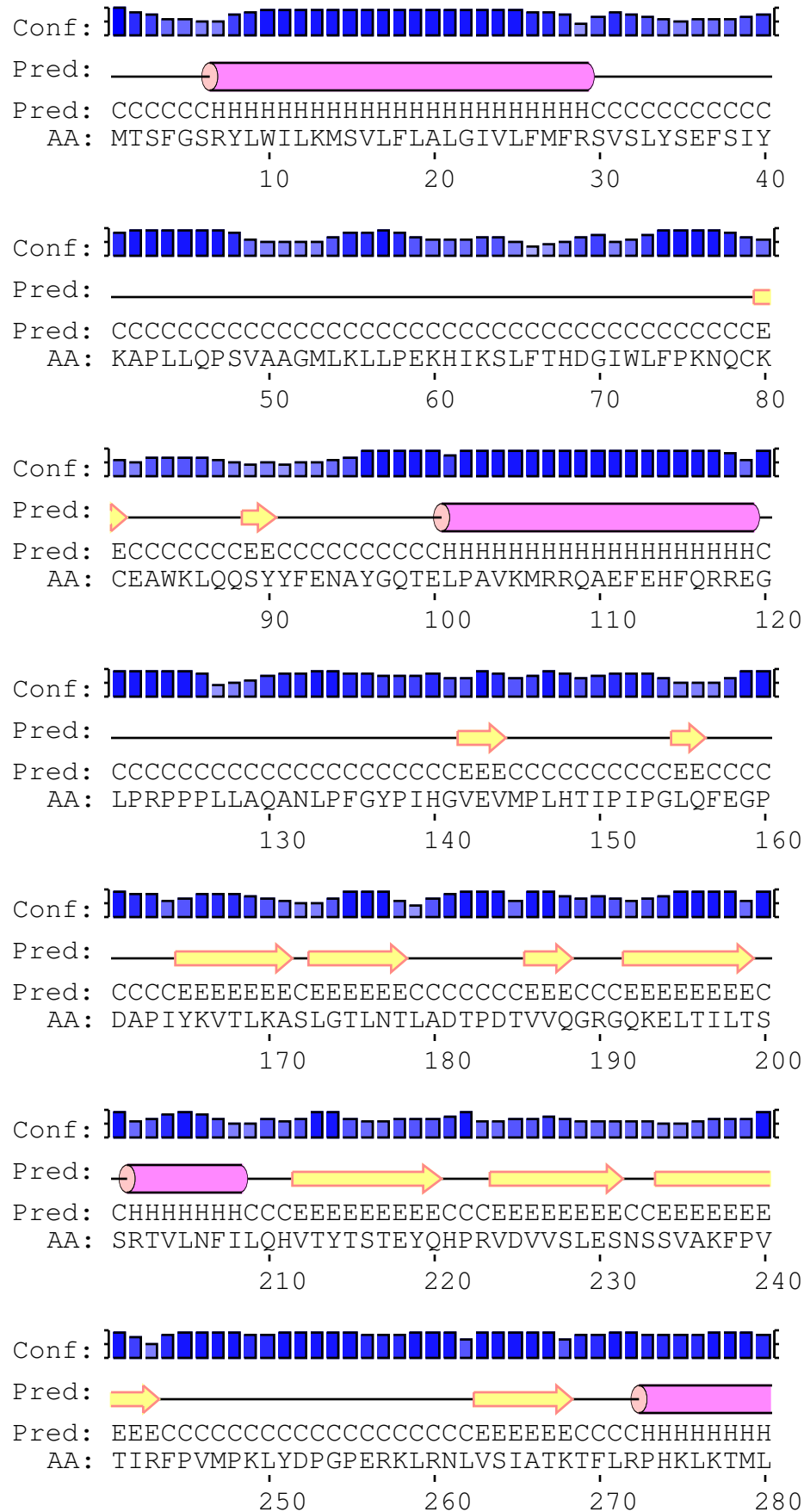

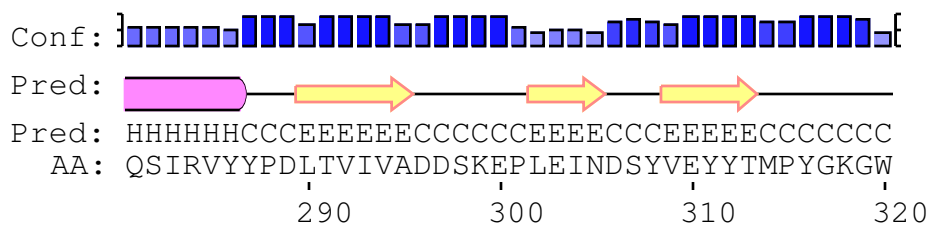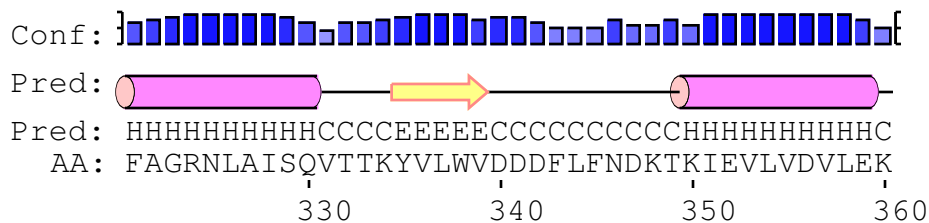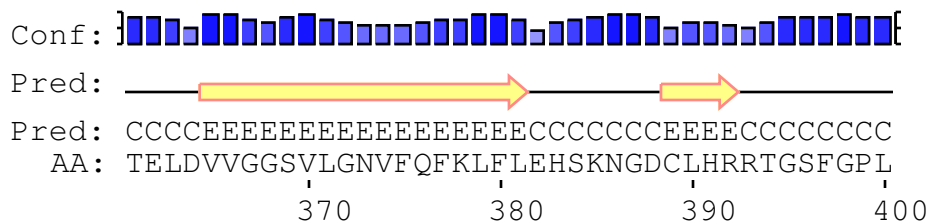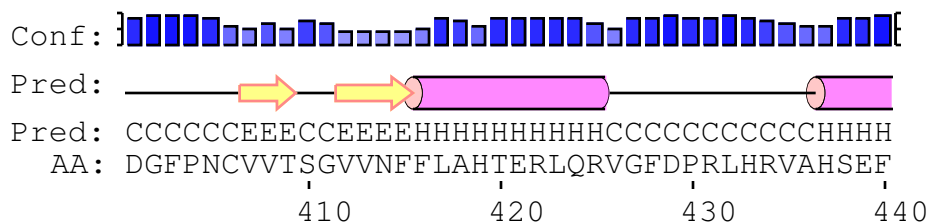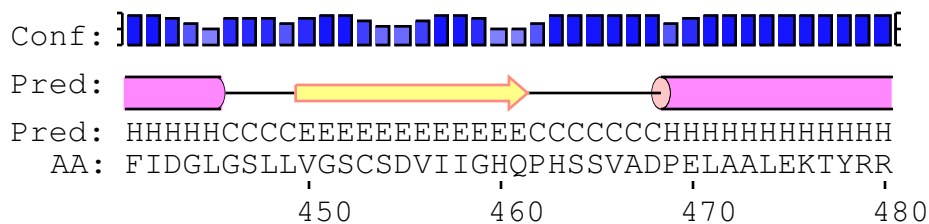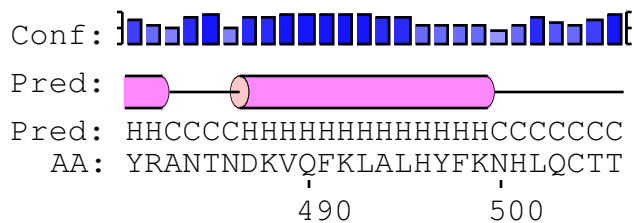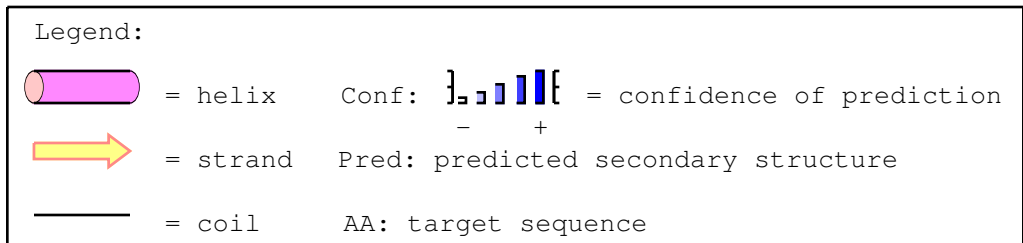

Supplement: Supplementary file 1 [file animals-08-00160-s001.zip › Supplementary file/Supplementary file 3.pdf]
